# Anti-HDV IgM as a Marker of Disease Activity in Hepatitis Delta

**DOI:** 10.1371/journal.pone.0101002

**Published:** 2014-07-29

**Authors:** Anika Wranke, Benjamin Heidrich, Stefanie Ernst, Beatriz Calle Serrano, Florin Alexandru Caruntu, Manuela Gabriela Curescu, Kendal Yalcin, Selim Gürel, Stefan Zeuzem, Andreas Erhardt, Stefan Lüth, George V. Papatheodoridis, Birgit Bremer, Judith Stift, Jan Grabowski, Janina Kirschner, Kerstin Port, Markus Cornberg, Christine S. Falk, Hans-Peter Dienes, Svenja Hardtke, Michael P. Manns, Cihan Yurdaydin, Heiner Wedemeyer, HIDIT-2 Study Group

**Affiliations:** 1 Department of Gastroenterology, Hepatology and Endocrinology, Hannover Medical School, Hannover, Germany; 2 Institute for Biometry, Hannover Medical School, Hannover, Germany; 3 Ankara University Medical Faculty, Ankara, Turkey; 4 Institutul de Boli Infectioase, Bucharest, Romania; 5 Spitalul Clinic de Boli Infectioase, Timisoara, Romania; 6 Dicle University Medical Faculty, Diyarbakir, Turkey; 7 Uludağ University Medical Faculty, Bursa, Turkey; 8 Johann Wolfgang Goethe University Medical Center, Frankfurt/Main, Germany; 9 Heinrich Heine University, Düsseldorf, Germany; 10 University Medical Centre Hamburg-Eppendorf, Hamburg, Germany; 11 Athens University School of Medicine, Athens, Greece; 12 Medical University of Vienna, Vienna, Austria; 13 Institute of Transplant Immunology, IFB-Tx, Hannover Medical School, Hannover, Germany; 14 HepNet Study-House, Hannover, Germany; 15 Integrated Research and Treatment Center Transplantation, Hannover Medical School, Hannover, Germany; 16 German Center for Infection Research (DZIF), Partner Side HepNet Study-House, Hannover, Germany; University of Washington, United States of America

## Abstract

**Background:**

Hepatitis delta frequently leads to liver cirrhosis and hepatic decompensation. As treatment options are limited, there is a need for biomarkers to determine disease activity and to predict the risk of disease progression. We hypothesized that anti-HDV IgM could represent such a marker.

**Methods:**

Samples of 120 HDV-infected patients recruited in an international multicenter treatment trial (HIDIT-2) were studied. Anti-HDV IgM testing was performed using ETI-DELTA-IGMK-2-assay (DiaSorin). In addition, fifty cytokines, chemokines and angiogenetic factors were measured using multiplex technology (Bio-Plex System). A second independent cohort of 78 patients was studied for the development of liver-related clinical endpoints (decompensation, HCC, liver transplantation or death; median follow up of 3.0 years, range 0.6–12).

**Results:**

Anti-HDV IgM serum levels were negative in 18 (15%), low (OD<0.5) in 76 (63%), and high in 26 (22%) patients of the HIDIT-2 cohort. Anti-HDV IgM were significantly associated with histological inflammatory (p<0.01) and biochemical disease activity (ALT, AST p<0.01). HDV replication was independent from anti-HDV IgM, however, low HBV-DNA levels were observed in groups with higher anti-HDV IgM levels (p<0.01). While high IP-10 (CXCL10) levels were seen in greater groups of anti-HDV IgM levels, various other antiviral cytokines were negatively associated with anti-HDV IgM. Associations between anti-HDV IgM and ALT, AST, HBV-DNA were confirmed in the independent cohort. Clinical endpoints occurred in 26 anti-HDV IgM positive patients (39%) but in only one anti-HDV IgM negative individual (9%; p = 0.05).

**Conclusions:**

Serum anti-HDV IgM is a robust, easy-to-apply and relatively cheap marker to determine disease activity in hepatitis delta which has prognostic implications. High anti-HDV IgM levels may indicate an activated interferon system but exhausted antiviral immunity.

## Introduction

Hepatitis delta is caused by infection with the hepatitis D virus (HDV) and represents the most severe form of chronic viral hepatitis [1]. Chronic hepatitis delta is associated with frequent development of liver cirrhosis, hepatic decompensation and hepatocellular carcinoma (HCC) [2]. HDV is a defective satellite virus that requires the help of the hepatitis B surface antigen for viral assembly and propagation [1]. Treatment options for hepatitis delta are limited. As HDV does not encode for a viral enzyme, no specific direct acting antivirals against HDV are available. Pegylated interferon alpha induces HDV-RNA negativity in about one quarter of patients [3], [4]. However, treatment is poorly tolerated with significant side effects in particular in patients with advanced liver disease [4]. In single patients treatment with interferon alpha can be even harmful. Biomarkers are therefore needed to predict the long-term outcome of hepatitis delta and to identify patients at most urgent need for therapy.

There is currently no reliable non-invasive marker associated with disease activity in hepatitis delta. Quantitative HDV-RNA levels do not correlate with grade or stage of liver disease in HDV-infected patients [5]. Quantitative HBsAg levels show some correlation with histological activity but associations are weak [5]. Similarly the HBeAg status is not associated with distinct outcomes in HDV-infected patients [6].

Anti-HDV Immunoglobulin M (IgM) testing was used to diagnose hepatitis delta infection before HDV-RNA assays became available [7]. Anti-HDV IgM can persist in chronic hepatitis delta patients and reappears in patients with relapse after therapy [8], [9], [10]. We previously showed in a smaller cohort of hepatitis delta patients that anti-HDV IgM levels may correlate with histological inflammatory activity [11]. Nevertheless, these findings were not yet reproduced in larger cohorts and the potential role of anti-HDV IgM testing to predict the clinical long-term outcome of hepatitis delta virus infection is unknown. Moreover detailed mechanisms on the immunopathogenesis of HDV infection leading to different anti-HDV IgM activities are largely undefined [12].

Our primary aim was, therefore, to investigate possible associations of anti-HDV IgM with grade and stage of liver disease in hepatitis delta in a cross-sectional approach testing very well characterized samples from a large multicenter study. In a second step, we investigated whether or not anti-HDV IgM activity can predict the clinical long-term outcome in hepatitis delta. Finally, we questioned if specific cytokines, chemokines and angiogentic factors were associated with anti-HDV IgM to understand possible mechanisms regulating humoral immunity against HDV.

## Methods

### 2.1. Patients

Two independent cohorts of patients were studied. First, we analyzed baseline data of the Hep-Net-International-Delta-Hepatitis-Intervention Trial-2 (HIDIT-2) an prospective international, multicentre trial, investigating the efficacy of PEG-IFN alfa-2a plus tenofovir or placebo for 96 weeks in 121 patients chronically infected with HDV (www.clinicaltrials.gov; NCT00932971; EudraCT-No.: 2008-005560-13). Patients with compensated liver disease (Child A) and absence of HIV or HCV coinfection were eligible. Liver biopsies were performed within one year prior baseline.

Central histological pathological reading was performed by two independent and blinded pathologists (H.P.D, J.S.). Grading and staging was performed according to the Ishak score [13]. All biopsies with an Ishak score of F5 or F6 were defined as cirrhosis. Additionally, cirrhosis was also diagnosed when biopsies did not confirm cirrhosis but two of the following criteria coexisted: platelet count below 100000/ml, AST/ALT ratio >1, bilirubin levels at least 1.5 times higher than upper limit of normal (ULN), Albumin <35 g/l or presence of varices.

A total activity index of 0–7 was considered as minimal or mild hepatitis, a score of 8–18 indicated severe hepatitis.

As the HIDIT-2 trial allowed only cross sectional investigation we recruited an additional independent cohort with patients seen at the Hannover medical school from 1995 to 2012 (“MHH cohort”). Of all anti-HDV IgM positive patients a cohort of 78 chronic hepatitis delta patients were screened to correlate liver-related endpoints with the anti-HDV IgM status. Liver-related endpoints were defined as defined as hepatic decompensation (ascites, encephalopathy, variceal bleeding), liver transplantation, HCC or liver-related death. Patients were included in this study if serum samples or anti-HDV IgM testing were available in addition to the following inclusion criteria. Patients were required to have detectable HBsAg and either anti-HDV IgM antibodies or HDV-RNA for at least six months. Only patients with an available follow up of at least 6 months with a minimum of 2 visits and no longer than 2 years between these visits were included. Patients were excluded if they had undergone liver transplantation, or suffered from HCC before the first observation. Virological parameters for hepatitis B and delta were measured as previously described [5], [14].

The MHH cohort was recruited from a real world setting and thus patients received various medications during further follow up including nucleoside and nucleotide analogues (n = 16), PEG- IFN alpha (n = 3) and combinations of interferon and HBV polymerase inhibitors (n = 13). 46 patients did not receive any antiviral therapy during follow-up (n = 46).

#### Ethics Statement

The HIDIT-2 study was obtained by the approving institutional review board of the Hannover Medical School (Ethikkommission der Medizinischen Hochschule Hannover) and the local ethic committees (Landesamt für Gesundheit und Soziales Berlin, Ethikkommission an der Medizinischen Fakultät der Heinrich-Heine-Universität Düsseldorf, Ethikkommission an der Medizinischen Fakultät der Rheinischen Friedrich-Wilhelms-Universität Bonn, Ethikkommission der J.W.-Goethe-Universität Frankfurt, Ethikkommission der Ärztekammer Hamburg, Ethikkommission der Medizinischen Fakultät der Ludwig-Maximillians-Universität, Ethikkommission der Medizinischen Fakultät der Universität Heidelberg, Ethikkommission der Medizinischen Fakultät der Universität Würzburg, Ethikkommission an der Medizinischen Fakultät der Universität Leipzig) (Nr. 5292M, EudraCT-Nr 2008-005560-13). All of the named ethic committees approved this study. For all of the patients informed consent paperwork was written.

For the measurement of cytokines, chemokines and angiogenetic factors the Ethikkommission der Medizinischen Hochschule Hannover had reviewed the experiments and approved this study design (Nr. 5258).

The internal use of data of the MHH cohort was reviewed by the Ethikkommission der Medizinischen Hochschule Hannover and considered that no informed consent was necessary, given the retrospective and noninterventional nature of the study, and that patient's data were analyzed anonymously.

### 2.2. Anti-HDV IgM

Anti-HDV IgM testing was performed by using ETI-DELTA-IGMK-2 assay (DiaSorin, Saluggia, Italy) according to manufacturer's instructions. In the first step samples were attached to monoclonal IgG directed at human IgM. After one hour of incubation and washing, HDAg was added. Staining was achieved by adding anti-HD horseradish peroxidase and in a last step chromogen to the sample dilution. The optical density (OD) 450/620 values were ascertained by using photometry (Tecan Rainbow Thermo, Tecan, Crailsheim, Germany).

All samples with an anti-HDV IgM OD 450/620 above the mean extinction of the negative control plus 0.1 were considered to be positive. Samples with optical density values of 10% below or above this cut-off were retested.

To investigate to what extent quantitative values of the assay could be used for further analyses we evaluated the performance of the assay with different sera and plasma samples. Sera and plasma samples obtained in parallel from same individuals revealed similar IgM in all individuals tested (data not shown).

We next investigated different incubation periods. While samples with low IgM levels (<0.5 OD) and very high levels (>2.5) gave similar values irrespective of the incubation period, a high variability was observed for samples with intermediate and high values (0.5–2.5) considering different incubation periods (Figure S1).

We therefore did not further consider evaluation of OD as linear variables for further investigations but rather grouped IgM levels into four categories (negative, <0.5, 0.5–2.5, >2.5).

### 2.3. Quantification of cytokines, chemokines and growth factors

The concentrations of fifty cytokines, chemokines and angiogenetic factors were measured in sera using the Luminex-base multiplex technology.

Bio-Plex assays (Bio-Rad, Hercules, USA) contain standard concentrations of each analyte and the calculation of respective standard curves allows a precise definition of the concentrations of the protein of interest. The assay was performed according to the manufacturer's protocol. In brief, lyophilized cytokine standard was resuspended in standard diluent. Serial dilution series to generate standard curves for each cytokine, chemokine and growth factor of interest were performed. The bead mixture, specific for cytokines, chemokines or growth factors was incubated for 30 min at RT with 50 µl standard or serum samples that were diluted in sample diluent (1∶2 dilution). Several washing steps were performed with 100 µl wash buffer/well, using the automated washer for magnetic beads. After addition of secondary biotinylated antibody mix for 30 min at RT and three more washing steps, Streptavidin-conjugated R-phycoerythrin (SAPE) was added for 10 min at RT (1∶100 dilution). After three final washing steps, beads were resuspended with 125 µl assay buffer, acquired and analyzed by the BioPlex Manager 6.0 software.

### 2.4. Statistics

Statistical analyses were performed by using SPSS software (SPSS Inc., Chicago, Illinois, USA).

All parameters were described as median. P-values <0.05 were considered as significant factors.

Parametric values were analysed by T-Test. For non-parametric (distribution-free) parameters Mann-Whitney-U-tests were used. A Fisher exact test was calculated for the comparison of discrete variables.

The association of IgM with liver related clinical endpoints was additionally identified by Cox-regression models and Kaplan- Meier analysis.

## Results

To investigate the role of anti-HDV IgM testing in hepatitis delta, two independent patient cohorts were studied. Baseline characteristics of patients in both cohorts are shown in Table 1. The HIDIT-2 cohort consisted of individuals fulfilling inclusion criteria for PEG-IFNα-based treatment, thus, individuals with decompensated liver disease were excluded. The second cohort included consecutive patients treated at a tertiary referral center as part of routine clinical follow-up. Both cohorts were similar in terms of gender distribution, mean age, biochemical disease activity and proportion of patients with cirrhosis. Patients in the MHH cohort had slightly more advanced liver fibrosis with a higher mean APRI-Score, higher AST-/ALT- ratios, lower mean albumin levels and lower platelet counts. In the HIDIT-2 cohort most of the patients originated from the Eastern Mediterranean area, whereas in the MHH cohort nearly the same percentage of patients was born in the Eastern Mediterranean area and Eastern Europe regions.

**Table 1:** Baseline characteristics.

	HIDIT-2	MHH cohort
*Total*	120	78
***Sex***	male = 79 (66%)	male = 49 (63%)
	female = 41 (34%)	female = 29 (37%)
***Age (years)*** * [median* (interquartile range)]	39.9 (31.8–49.6) (n = 120)	40 (30.1–48.8) (n = 78
***Country of origin***	Eastern Mediterranean = 68 (57%)	Eastern Mediterranean = 27 (35%)
	Eastern Europe = 44 (37%)	Eastern Europe = 31 (40%)
	Italy = 1 (1%)	Italy = 4 (5%)
	Central Europe = 3 (2%)	Central Europe = 6 (8%)
	other = 4 (3%)	other = 10 (12%)
***Anti-HCV positive***	0	11 (14.1%)
***AST (x ULN)*** * [median (interquartile range)]*	1.6 (1.1–2.3) (n = 119)	1.9 (1.6–2.6) (n = 75)
***ALT (x ULN)*** * [median (interquartile range)]*	2.0 (1.3–3.) (n = 119)	1.9 (1.0–3.0) (n = 74)
***AP (x ULN)*** * [median (interquartile range)]*	0.6 (0.5–0.7) (n = 119)	0.8 (0.6–1.1) (n = 72)
***γGT (x ULN)*** * [median (interquartile range)]*	0.8 (0.6–1.5) (n = 118)	0.8 (0.6–1.7) (n = 75)
***Bilirubin (µmol/L)*** * median (interquartile range)]*	11.4 (8.6–17.1) (n = 116)	11.0 (8.0–20.0)(n = 71)
***Albumin (g/L)*** * [median (interquartile range)]*	42.0 (39.0–45.0) (n = 119)	39 (35.0–42.0) (n = 63)
***Platelets (1000/µL)*** * [median (interquartile range)]*	172.0 (130.0–208.0) (n = 119)	130.5 (77.3–165.5) (n = 74)
***INR*** * [median (interquartile range)]*	1.1 (1.0–1.2) (n = 115)	1.1 (1.1–1.2) (n = 70)
***APRI-Score*** * [median (interquartile range)]*	1.0 (0.9–1.6) (n = 119)	1.7 (1.0–3.1) (n = 74)
***AST/ALT-Ratio*** * [median (interquartile range)]*	0.7 (0.6–0.8) (n = 119)	0.9 (0.6–1.2) (n = 73)
***BEA-Score*** * [median (interquartile range)]*	2.0 (1.0–3.0) (n = 109)	2.0 (1.0–3.0) (n = 77)
***MELD*** * [median (interquartile range)]*	7.5 (6.8–8.2) (n = 112)	8.0 (7.1–9.3) (n = 66)
***Child-Pugh classes***	A = 112 (93%)	A = 51 (65%)
	B = 0	B = 8 (10%)
	C = 0	C = 1 (1%)
***Cirrhosis***	51 (43%) (n = 120)	33 (42%) (n = 78)

### High frequency of anti-HDV IgM in patients with chronic hepatitis delta

**Figure 1:**
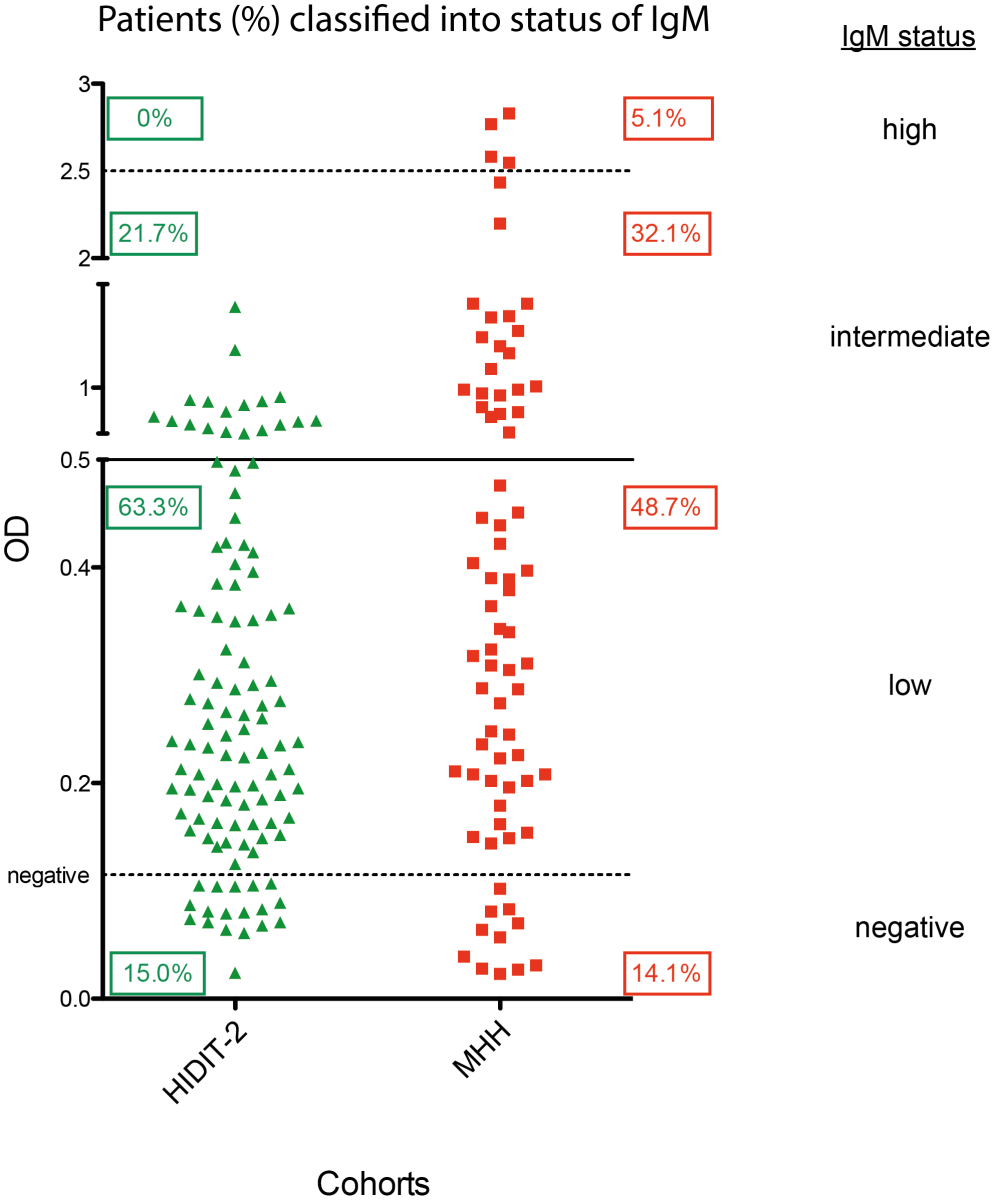
Anti-HDV IgM values in the two cohorts. Grouping into different IgM categories was performed as descripted in materials and methods.

Anti-HDV IgM tested positive in 102 patients (85%) of the HIDIT-2 cohort and in 67 patients (85.9%) of the MHH cohort. 63% of patients in the HIDIT-2 cohort and 49% of patients in the MHH cohort presented with low anti-HDV IgM levels. More patients in the MHH cohort showed intermediate IgM values (32%) or even high IgM levels (5%) than in the HIDIT-2 cohort (21% and 0%, respectively) (Figure 1).

### Anti-HDV IgM levels are associated with disease activity in hepatitis delta

**Figure 2:**
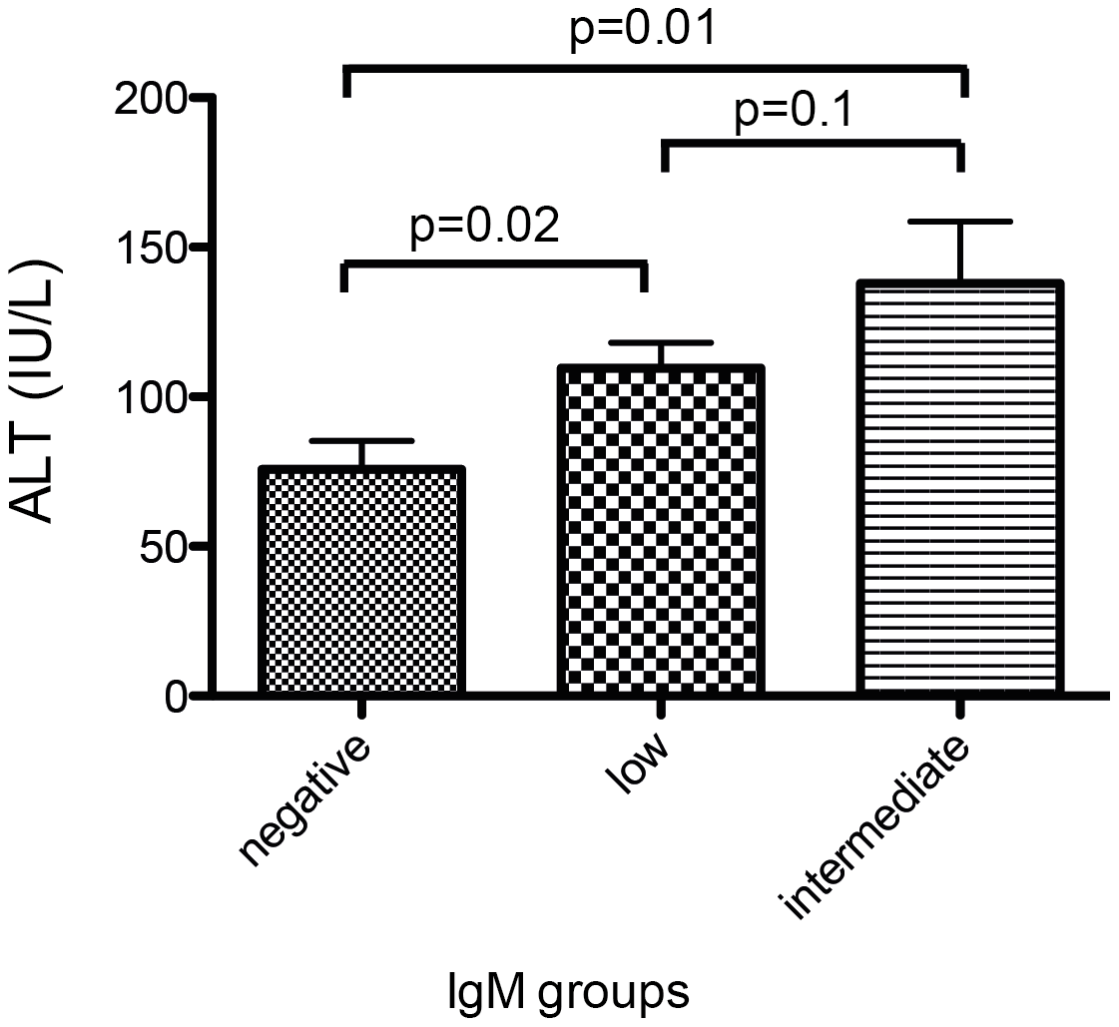
Distribution of ALT values according to Anti-HDV IgM categories in the HIDIT-2 cohort. ALT values were significantly associated with the different anti-HDV IgM groups based on univariate ANOVA (p = 0.04) and univariate T- test in the HIDIT-2 cohort.

**Figure 3:**
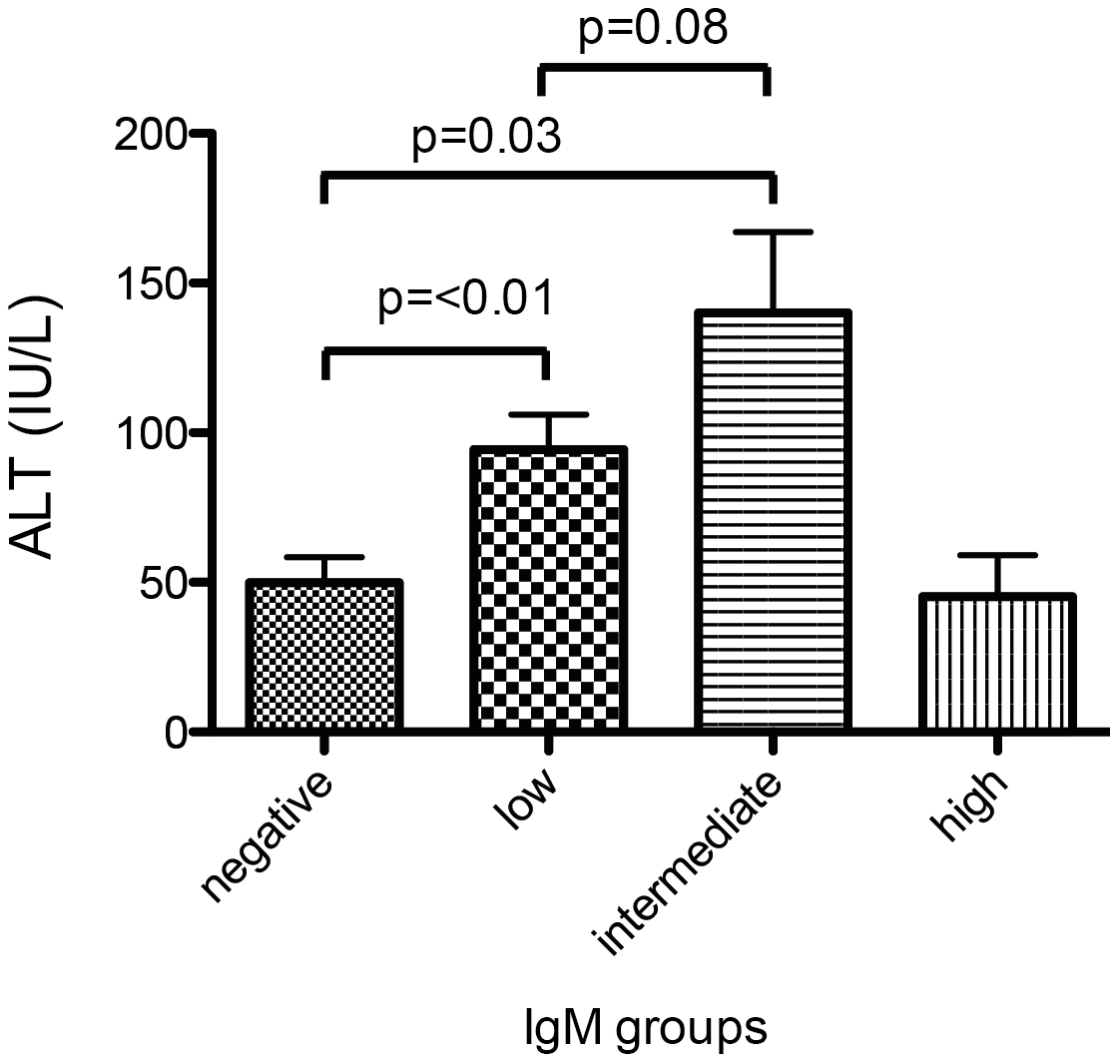
Distribution of ALT values according to Anti-HDV IgM categories in the MHH cohort (only significant p-values). The association of anti-HDV IgM and ALT was confirmed in the MHH cohort with significance over all groups of 0.03.

**Figure 4:**
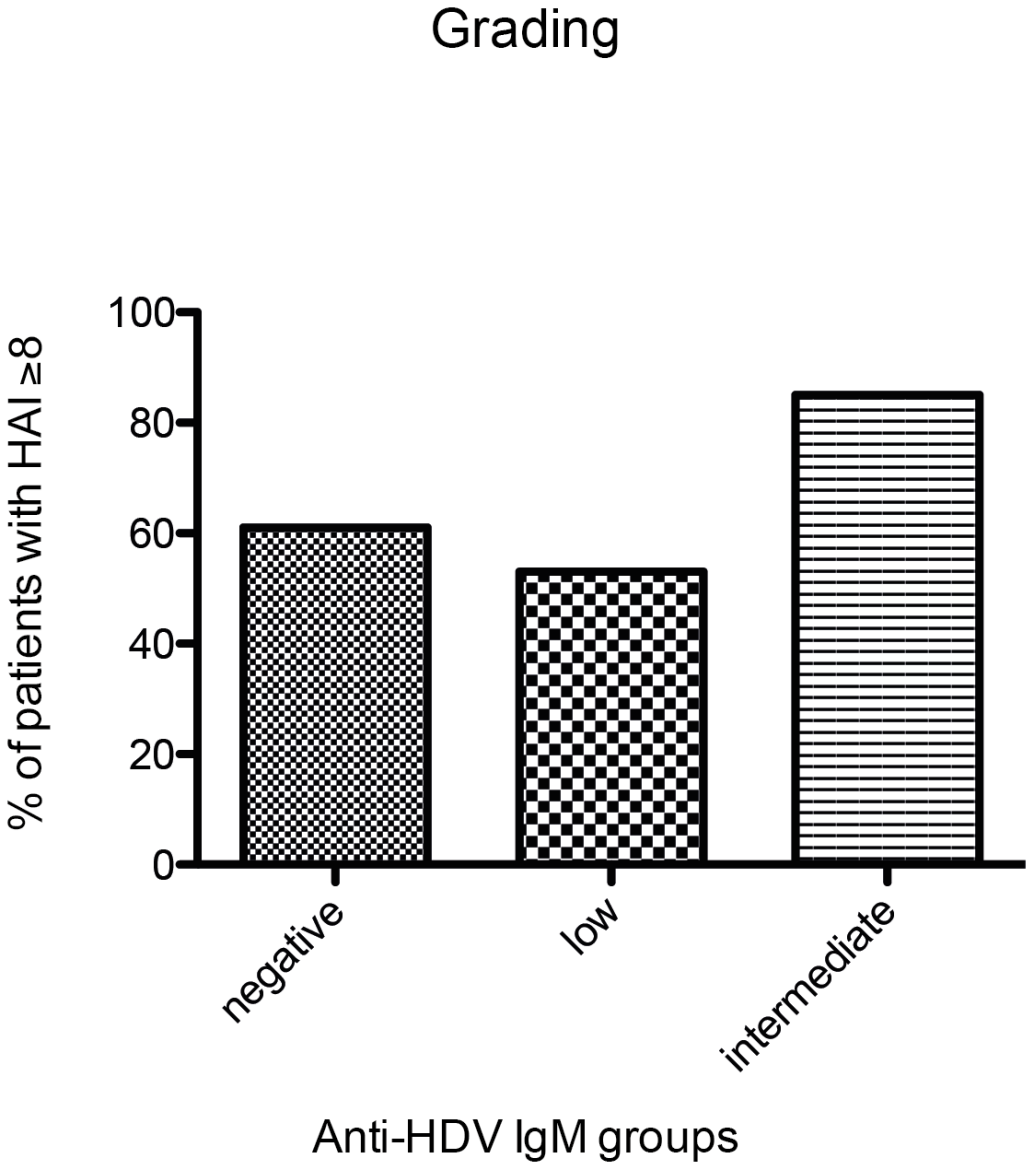
Histological grading of the HIDIT-2 cohort. Diagrammed% of patients with a HAI≥8 within the anti-HDV IgM groups.

**Figure 5:**
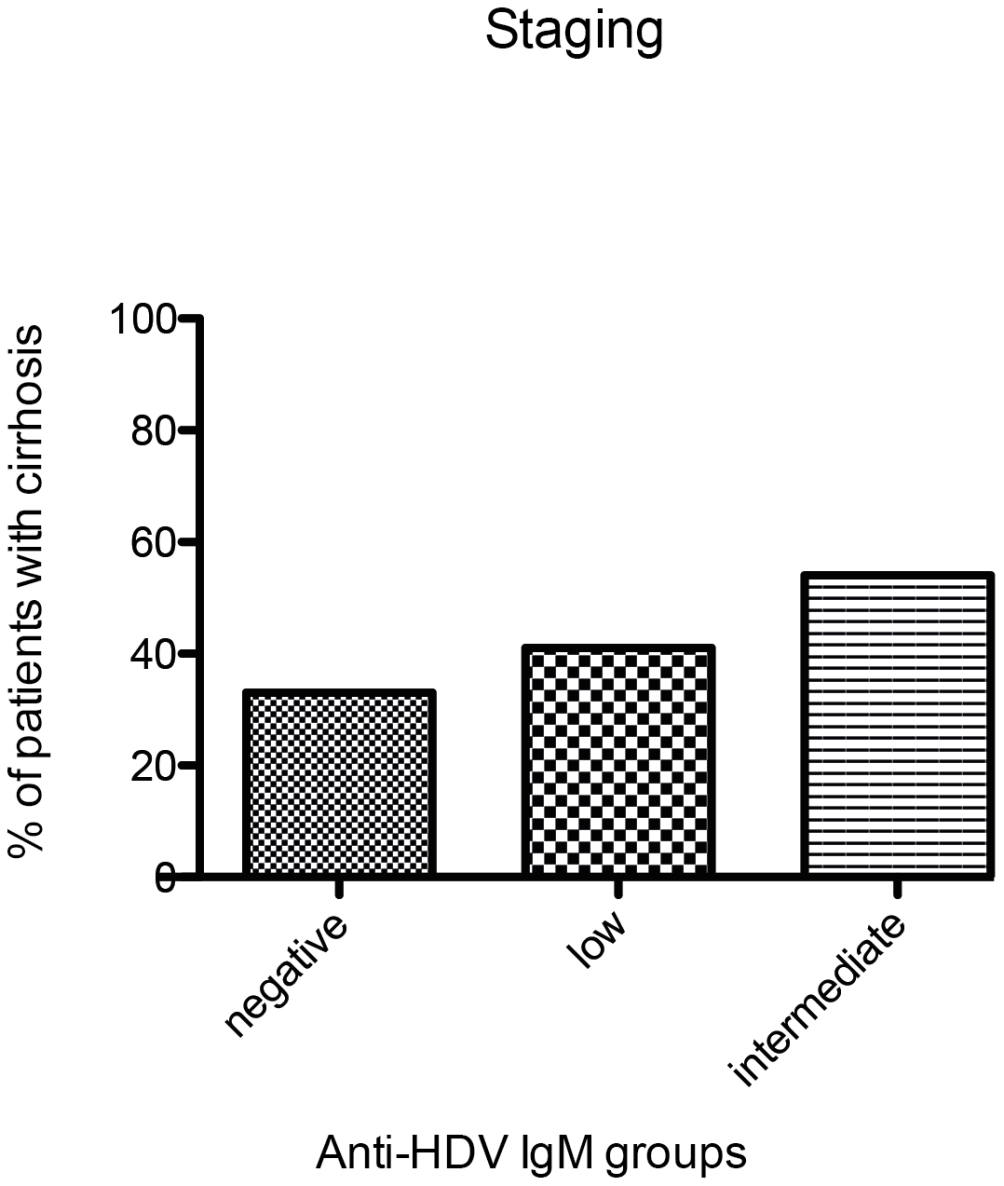
Histological staging of the HIDIT-2 cohort. Diagrammed% of patients with cirrhosis (based on the classification indicated in material and methods) within the anti-HDV IgM groups.

We next investigated if different categories of anti-HDV IgM levels were associated with clinical, biochemical or virological markers of liver disease in hepatitis delta. As shown in Figures 2 and 3, ALT levels were significantly associated with anti-HDV IgM levels in both cohorts. Similarly, anti-HDV IgM positive individuals had more frequently higher AST levels, higher bilirubin values and lower albumin levels than anti-HDV IgM negative patients in the HIDIT-2 cohort (Table 2). These differences were confirmed for AST and albumin in the MHH cohort (Table 3). Anti-HDV IgM levels were also associated with histological activity when comparing a total histological activity index of 0–7 vs. ≥8 by chi-square analysis (p = 0.02) (Figure 4). In contrast, histological staging was not associated with anti-HDV IgM patients when patients were grouped into cirrhotic and non-cirrhotic individuals (p = 0.4) (Figure 5).

**Table 2:** Factors associated with anti-HDV IgM positive and negative values according to chi-square analysis in the HIDIT-2 cohort.

HIDIT-2			
	IgM positive	IgM negative	*p* (χ^2^-test)
*Total*	n = 102	n = 18	
***AST (> ULN)*** * [n patients (median AST levels [U/l])*]	n = 63 (116.5)	n = 7 (75.7)	0.06
***ALT (> ULN)*** * [n patients (median ALT levels [U/l])*]	n = 74 (78.3)	n = 11 (55.2)	0.2
***γGT (> ULN)*** * [n patients (median γGT levels [U/l])*]	n = 41 (73.0)	n = 7 (72.2)	0.6
***Bilirubin (> ULN)*** * [n patients (median bilirubin levels [µmol/L])*]	n = 29 (13.8)	n = 1 (10.7)	0.03
***Albumin (< LLN*** * [n patients (median albumin levels [g/L])*]	n = 3 (42.2)	n = 3 (40.1)	0.04
***Platelets (< 100000/µL)*** * [n patients (median platelets levels [µL])*]	n = 13 (177 727)	n = 0 (174 056)	0.1
***HDV-RNA (<20000, 20000–200000, >200000)*** * [n patients (median HDV-RNA levels [IU/m ])*]	n = 24; 34; 38 (4.9)	n = 5;7; 6 (4.6)	0.9
***HBV-DNA (<20000, 20000–200000, >200000)*** * [n patients (median HDV-RNA levels [IU/ml])*]	n = 53; 11; 1 (2.4)	n = 6; 5; 3 (3.6)	<0.01
***HBsAg (<1000, 1000–10000, >10000)*** * [n patients (median HDV-RNA levels [IU/ml])*]	n = 6; 51; 42 (3.9)	n = 1; 7; 10 (4.0)	0.6

Listed bracketed are the median values of the parameters. Virological values are listed in log.

* LLN: lower limit of normal. **ULN**: upper limit of normal.

**Table 3:** Factors associated with anti-HDV IgM positive and negative values according to chi-square analysis in the MHH cohort.

MHH cohort*			
	IgM positive	IgM negative	*p* (χ^2^-test)
*Total*	n = 67	n = 11	
***AST (> ULN)*** * [n patients (median AST levels [U/l])*]	n = 54 (98.8)	n = 5 (44.9)	<0.01
***ALT (> ULN)*** * [n patients (median ALT levels [U/l])*]	n = 45 (108.0)	n = 4 (50.0)	0.03
***γGT (> ULN)*** * [n patients (median γGT levels [U/l])*]	n = 29 (82.0)	n = 2 (32.1)	0.09
***Bilirubin (> ULN)*** * [n patients (median bilirubin levels [µmol/L])*]	n = 20 (18.0)	n = 4 (13.7)	0.5
***Albumin (< LLN)*** * [n patients (median albumin levels [g/L])*]	n = 21 (37.9)	n = 1 (41.1)	0.09
***Platelets (< 100000/µL)*** * [n patients (median platelets levels [µL])*]	n = 24 (130 126)	n = 2 (162 363)	0.2
***HDV-RNA (<20000, 20000–200000, >200000)*** * [n patients (median HDV-RNA levels [IU/ml])*]	n = 48; 6; 12 (4.9)	n = 9; 2; 0 (4.0)	0.2
***HBV-DNA (<20000, 20000–200000, >200000)*** * [n patients (median HDV-RNA levels [IU/ml])*]	n = 28; 2; 1 (2.7)	n = 3; 1; 0 (3.1)	0.4
***Endpoints***	n = 26	n = 1	0.05

Listed bracketed are the median values of the parameters. Virological values are listed in log.

* LLN: lower limit of normal. **ULN**: upper limit of normal.

*HBsAg levels were available only for a subgroup of patients and could therefore not be analysed.

**Figure 6:**
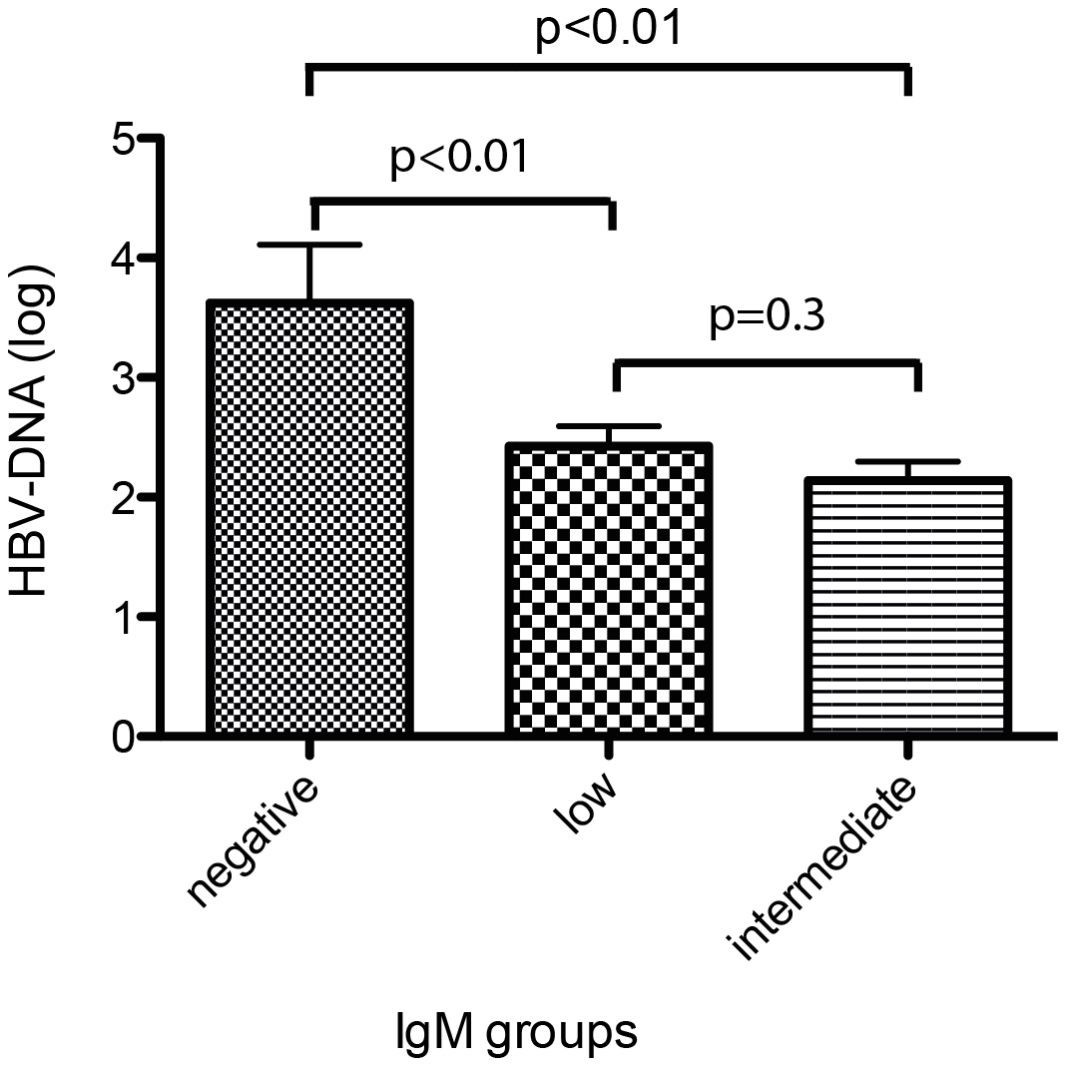
Distribution of HBV values according to Anti-HDV IgM categories in the HIDIT-2 cohort. HBV-DNA (log) is clearly associated with anti-HDV IgM indicated by ANOVA (p<0.01) and by T-test during the different IgM groups.

**Figure 7:**
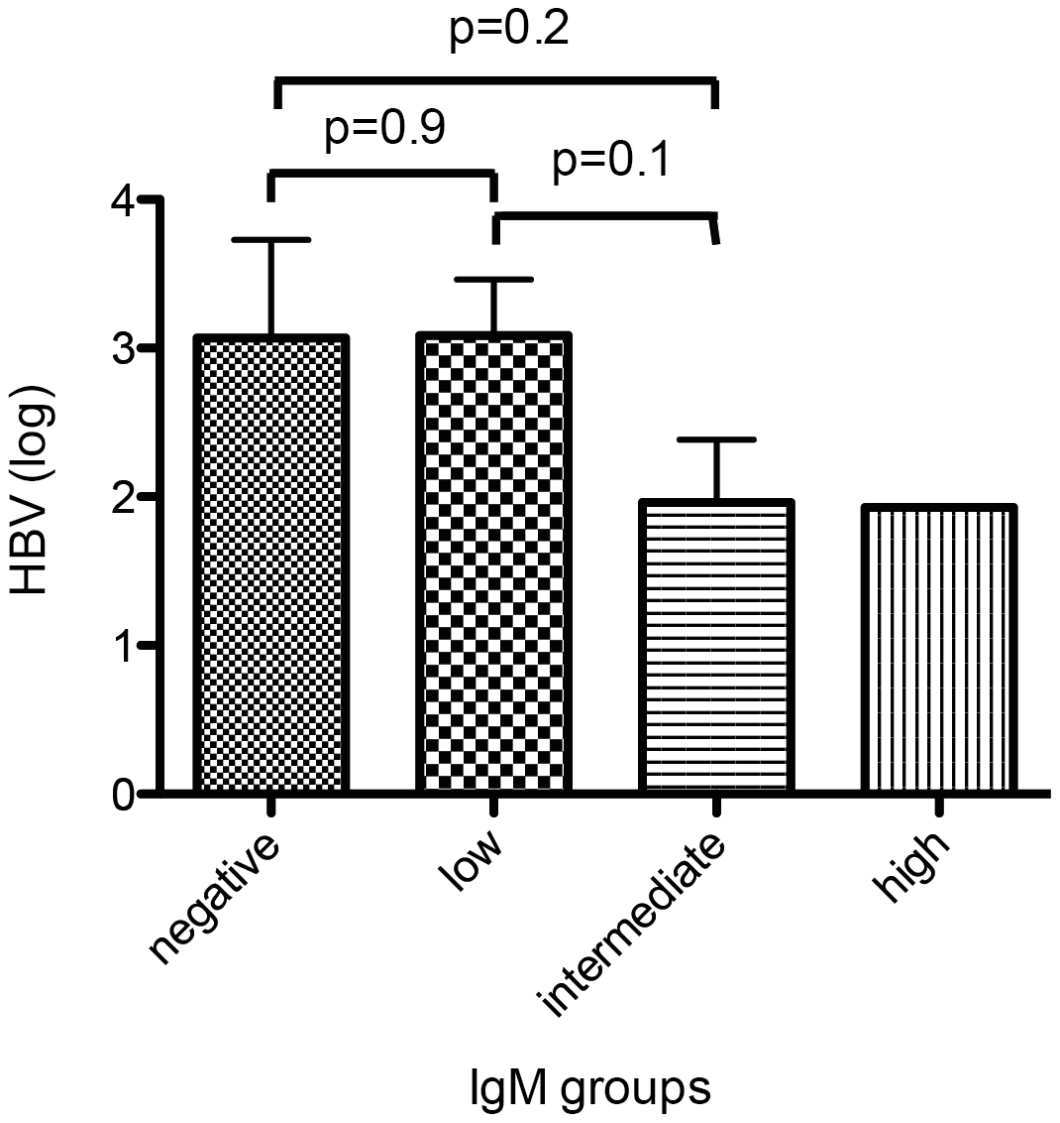
Distribution of HBV values according to Anti-HDV IgM categories in the MHH cohort. HBV-DNA levels were also lower in greater anti-HDV IgM groups in the MHH cohort although no differences could be observed within the anti-HDV IgM groups.

HDV-RNA levels, analyzed both as linear log-transformed values as well as grouped into low, median or high viremia, did not show any association with anti-HDV IgM categories in either of the cohorts (Table 2). Similarly, quantitative HBsAg levels were independent from anti-HDV IgM levels (Table 2). However, HBV-DNA levels were significantly higher in anti-HDV IgM negative patients in the HIDIT-2 cohort (Tables 2 & 3, Figure 6). In the MHH cohort HBV-DNA levels were only available for 35 patients (45%) (anti-HDV IgM negative:4; low:19; intermediate:7; high:1)The differences within the anti-HDV IgM groups were not significant. Nevertheless, the analysis indicated high HBV-DNA levels were more frequently seen in anti-HDV IgM negative patients or in patients with low anti-HDV IgM status (Figure 7).

Thus, high anti-HDV IgM groups were associated with biochemical and histological activity of liver disease and with lower HBV-DNA levels but were independent from HDV replication.

### Distinct cytokine and chemokine profiles are associated with anti-HDV IgM levels

To investigate if certain serum inflammatory patterns are associated with anti-HDV IgM levels, a large set of cytokines, chemokines and angiogenic factors was determined in plasma by multipex protein arrays (Table S1). Patients with intermediate anti-HDV IgM levels displayed lower serum levels of various pro-inflammatory cytokines including interleukin (IL-) 1, IL- 17, soluble interleukin-2 receptorα (sCD25) and chemokines like IL-8 (CXCL8), SDF-1α (CXCL12) than anti-HDV IgM negative patients (Table 4). The only chemokine that was positively but not negatively associated with anti-HDV IgM was the interferon gamma-induced protein 10 (IP-10, CXCL10).

**Table 4:** Factors univariatly associated with the groups of anti-HDV IgM based on ANOVA analysis.

	*IgM groups*			*ANOVA*
	Negative	<0.5	0.5 - 2.5	*p-value*
**ALT (IU/L)** *[median* ± standard deviation]	75.7±40.3	109.5±74.2	137.9±103.5	0.04
**AST (IU/L)** *[median* ± standard deviation]	55.2±22.6	73.4±42.3	93.0±54.7	0.02
**HBV-DNA (log)** *[median* ± standard deviation]	3.6±2.1	2.4±1.2	2.4±0.7	<0.01
**IL-1β (pg/ml)** *[median* ± standard deviation]	5.2±18.6	0.6±1.5	0.1±0.1	0.05
**IL-1α (pg/ml)** *[median* ± standard deviation]	7.3±25.5	0.7±0.5	0.7±0.4	0.05
**IL-8/CXCL8 (pg/ml)** *[median* ± standard deviation]	20.01±1.44	8.50±3.50	7.81±3.02	0.04
**IL-17 (pg/ml)** *[median* ± standard deviation]	115.5±281.2	38.3±18.5	36.7±15.6	0.04
**IP-10/CXCL10 (pg/ml)** *[median* ± standard deviation]	468.3±202.4	856.5±528.8	1019.0±759.4	0.02
**MCP-1/CCL2 (pg/ml)** *[median* ± standard deviation]	20.9±23.1	14.0±8.4	11.3±6.4	0.04
**M-CSF (pg/ml)** *[median* ± standard deviation]	23.1±79.7	2.1±3.0	2.6±2.5	0.05
**IL-2Rα/sCD25 (pg/ml)** *[median* ± standard deviation]	620.2±1244.3	134.8±63.8	140.2±56.5	<0.01
**IL-16 (pg/ml)** *[median* ± standard deviation]	221.7±329.1	114.1±50.2	136.6±91.3	0.03
**LIF (pg/ml)** *[median* ± standard deviation]	63.9±228.1	2.0±3.4	2.1±4.3	0.04
**SCF (pg/ml)** *[median* ± standard deviation]	116.4±220.9	51.6±26.6	51.7±24.1	0.03
**SDF-1α/CXCL12 (pg/ml)** *[median* ± standard deviation]	1358.5±4664.1	54.5±31.7	59.4±28.2	0.03
**TNF-β (pg/ml)** *[median* ± standard deviation]	29.5±107.9	1.1±2.3	1.2±1.5	0.05
**b-NGF (pg/ml)** *[median* ± standard deviation]	6.9±19.9	1.6±0.8	1.9±0.7	0.05

**Figure 8:**
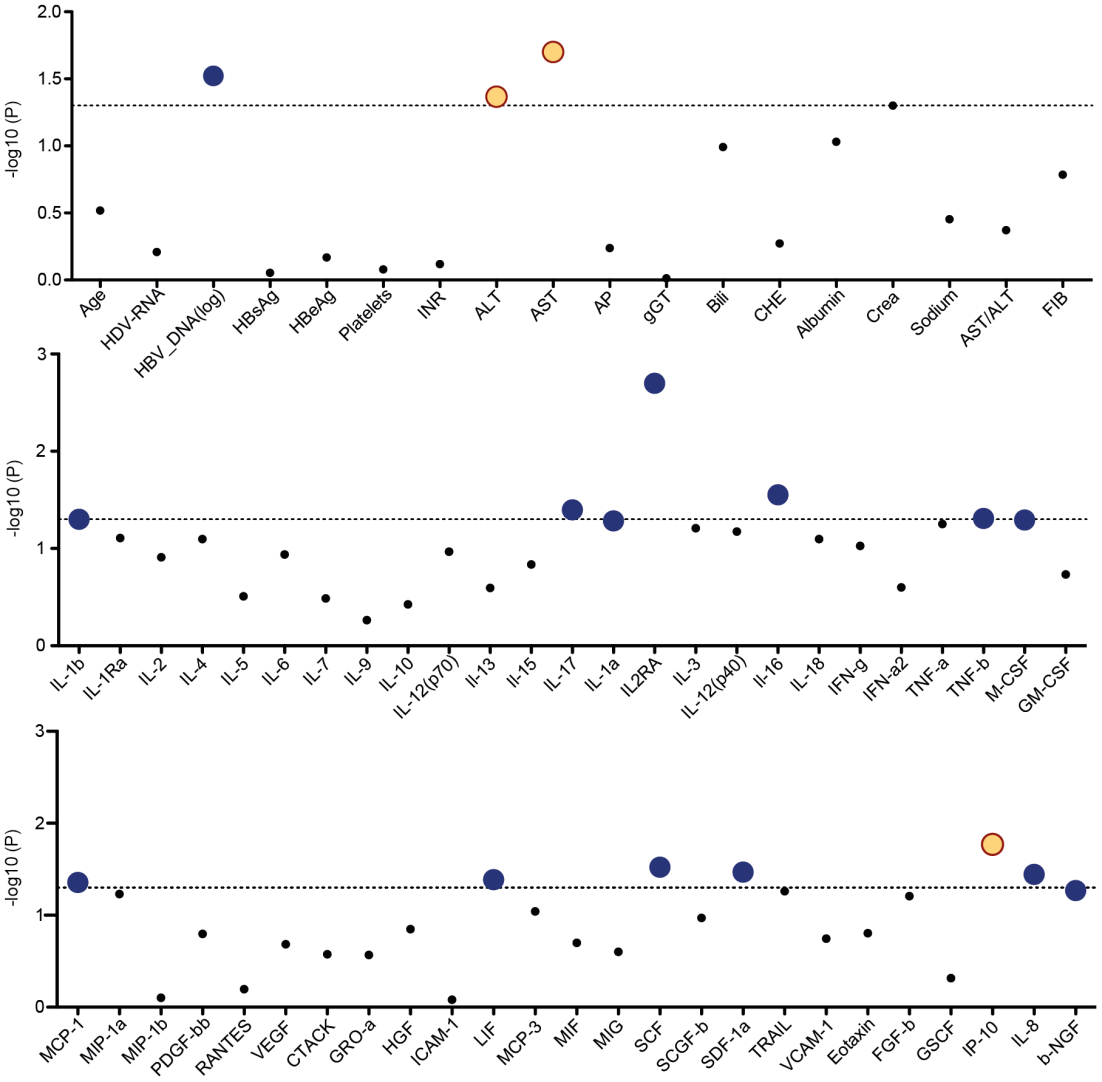
Factors associated with anti-HDV IgM based on univariate ANOVA testing in the HIDIT-2 cohort. The dashed line indicated a p-value of 0.05. All parameters above the line were determined as significantly associated with the four anti-HDV IgM groups. Dots in blue indicate low levels of the value in greater anti-HDV IgM groups while orange dots represent parameters with high levels in a great anti-HDV IgM group shown at x axis.

To illustrate the univariate ANOVA analysis of all measured parameters we developed a figure indicating which parameters were associated with the three anti-HDV IgM groups in the HIDIT-2 cohort and marked parameters that were high in the intermediate IgM group or low in the low IgM group (Figure 8).

### Anti-HDV IgM negative patients have a more benign clinical course of hepatitis delta

**Figure 9:**
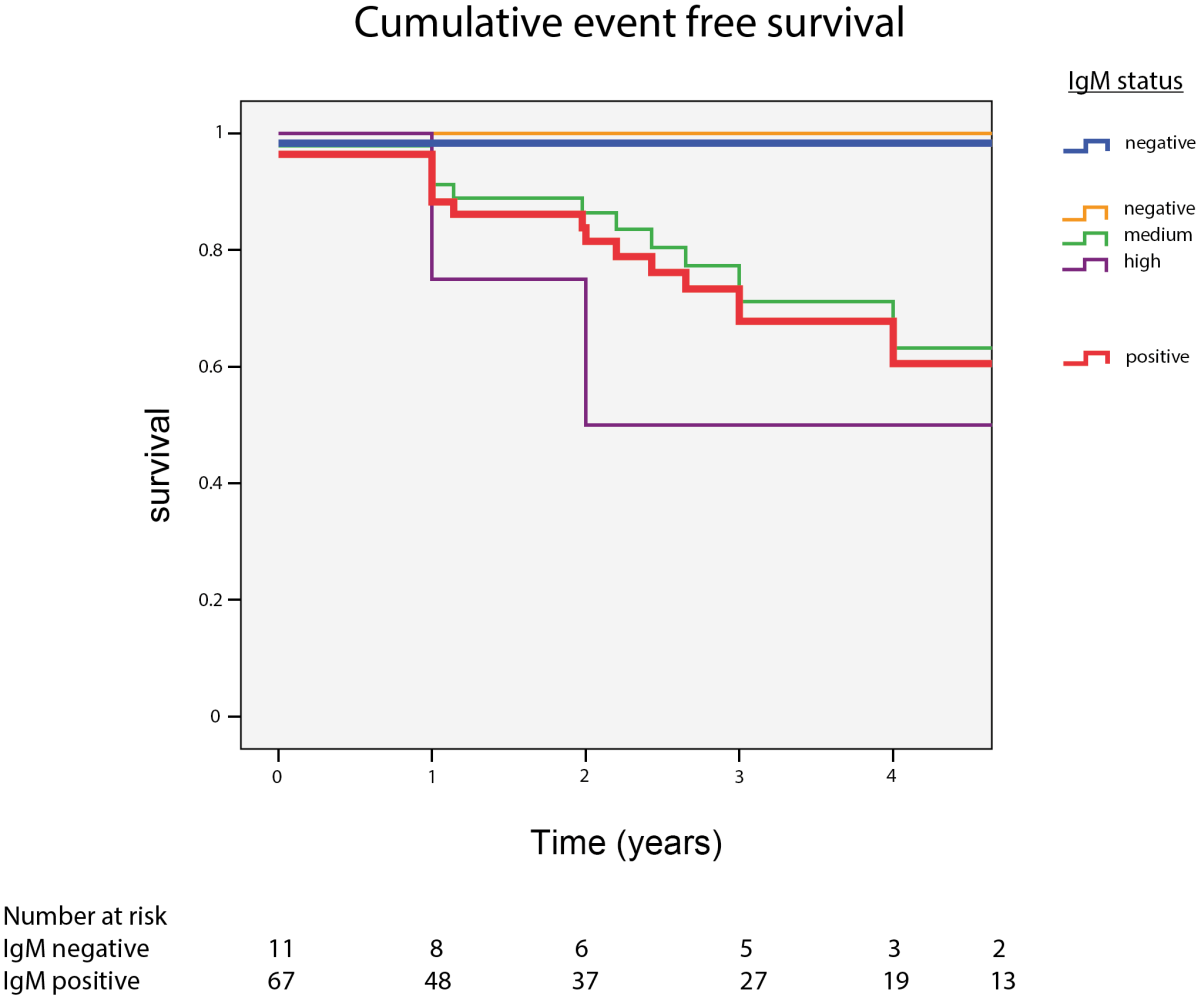
Cumulative event free survival of patients with anti-HDV IgM positive and negative values.

Clinical long-term outcome was investigated in 78 patients who were followed for up to 14 years (median 3 years; range 0.5–14.2). A liver-related clinical decompensation occurred only in one anti-HDV IgM negative patient (9%) but in 26 patients (39%) with positive IgM levels (p = 0.05). In addition to the Fisher exact test (Table 3), endpoints also showed a separation in Kaplan Meier analysis (Figure 9), even though the log-rank (Cox-regression) did not reach a significant value (p = 0.095). Different levels of anti-HDV IgM antibodies (low/high) were not associated with different clinical courses. Of note, the clinical long-term outcome was independent from treatment interventions and response to subsequent antiviral therapies (p = 0.6) or previous exposure to antiviral compounds (p = 0.3) (data not shown).

Thus, anti-HDV IgM testing may identify a subgroup of hepatitis delta patients with a very mild clinical course.

## Discussion

Hepatitis delta is the most severe form of chronic viral hepatitis. Considering the limited efficacy and the significant side effect profile of PEG-IFNα therapy, biomarkers are needed to distinguish patients with a more benign natural course of liver disease from individuals who have a higher risk to experience clinical decompensation. We here suggest that anti-HDV IgM may represent such a marker. IgM levels were associated with histological disease activity and, maybe even more importantly, the absence of anti-HDV IgM identified a subgroup of patients with a very mild course of liver disease that may not require immediate antiviral therapy.

The presence of IgM antibodies against a viral pathogen is usually considered to be associated with early acute infections. However, it is well established for various viral infections including hepatitis B that virus-specific IgM immunogloublins can be detected during all phases of the infection. E.g., anti-HBc IgM antibodies have been observed during flares of chronic hepatitis B [15]. Similarly, increasing levels of anti-HCV IgM have been associated with recurrent hepatitis C after liver transplantation [16]. In chronic hepatitis delta, anti-HDV IgM levels correlated with disease activity and treatment outcome already in earlier studies performed in the 1980ies and early 1990ies [8],[8], [10]. However, these study cohorts were rather small and no systematic blinded pathological reading was applied. The present study represents the so far largest cohort of hepatitis delta patients that has been tested for anti-HDV IgM. Another particular strength was that samples were obtained from a very well defined cohort of patients recruited in an international multicenter trial with monitored data and central pathological reading by an experienced pathologist who had no access to other clinical or immunological data. Moreover, a second independent “real-world” cohort of patients collected during routine clinical practice could be studied. With this data set we here demonstrate a rather high frequency of patients testing positive for anti-HDV IgM with more than 80% of patients showing different levels of IgM antibodies in both cohorts. This finding is well in line with our previous sub-analysis of 30 patients treated within the HIDIT-1 study [11].

The clinical value of anti-HDV IgM testing in patients with chronic hepatitis delta may be to identify patients with more advanced disease activity. Indeed, ALT and AST levels were significantly higher in patients with intermediate and high anti-HDV IgM levels. More importantly, patients with intermediate anti-HDV IgM levels had more frequently a high inflammatory activity than patients with low levels of or negative anti-HDV IgM. Remarkably, this association with disease activity also translated in differences in the clinical long-term outcome. Only one anti-HDV IgM-negative patient developed a clinical decompensation in the MHH cohort after a follow-up time of up to 12 years. Thus, in clinical practice, the decision to start a potentially toxic treatment based on PEG-IFNα could possibly be delayed in this subgroup of patients which represented around 15% of the cohorts analyzed in the current study. We do not believe that delaying therapy in anti-HDV IgM negative cases would reduce the chance to respond to PEG-IFNα therapy as our preliminary data from another international treatment trial indicated that lower anti-HDV IgM levels maybe associated with weaker but not better treatment responses [11].

Even though the specificity of a negative test to predict a benign clinical course was very high, the sensitivity of anti-HDV IgM testing concerning this particular read-out is still very limited. Grouping patients into low and intermediate anti-HDV IgM levels did not allow to further distinguish different clinical courses of hepatitis delta and thus additional biomarkers would be needed to identify more patients in which treatment may be safely deferred.

Anti-HDV IgM levels were strongly associated with serum CXCL10 (IP-10) levels. CXCL10 has recently been investigated in other hepatitis virus infections and was proposed as a serum marker indicating the activation status of the interferon system. A possible link between serum CXCL10 and anti-HDV IgM could be that IFNα induces CXCL10 and stimulates plasma cells which, thereby, possible leads to an increase anti-HDV IgM levels [17], [18]. We previously demonstrated that high levels of CXCL10 can be detected in the serum of hepatitis delta patients and that levels of CXCL10 further increased during PEG-IFNα therapy [19]. High CXCL10 levels have been associated with weaker responses to therapy in hepatitis C virus infection [20], [21], while, in contrast, higher CXCL10 levels correlated with more pronounced declines of HBsAg during polymerase inhibitor therapies in hepatitis B [22] and also PEG-IFNα-based HBV treatment [23]. In line with these findings, we here also describe that patients in a greater anti-HDV IgM group had high levels of CXCL10 levels and lower HBV-DNA values indicating suppressed HBV replication. However, serum HDV-RNA levels were not linked with anti-HDV IgM or serum CXCL10 levels. Thus, these data may suggest that HDV is controlled to a lesser extent by an activated interferon system than HBV. This observation could also partly explain why HDV is usually dominant over HBV as shown by numerous previous studies [5], [24], [25]. Nevertheless, these findings have to be tested in further experimental studies considering the cellular immunity against HDV.

In contrast to serum CXCL10, all other proinflammatory cytokines investigated in this study showed higher levels when anti-HDV IgM was negative. Thus, controlled HDV infection as indicated by lower disease activity and absence of anti-HDV IgM is associated with a functional broad proinflammatory immune response, indicated by high levels of these cytokines, while more severe disease seems to lead to exhaustion of the antiviral immune defence. Indeed, HDV-specific T cell responses are rather weak in chronic hepatitis delta with active disease [26] but future studies need to investigate this hypothesis in more detail.

Our study has obvious limitations. The clinical course of liver disease in the MHH cohort may have been altered by treatment interventions even though antiviral therapies were not associated with clinical long-term outcome. Moreover, future studies need to investigate anti-HDV IgM dynamics over time, which may even increase the value of IgM testing to predict the clinical outcome. Furthermore, anti-HDV IgM should be investigated in more detail during antiviral therapy. The anti-HDV IgM assay used here allowed reliable determination of semi-quantitative IgM levels; however, improved assays with a broader linear range would be needed to analyse anti-HDV IgM as a continuous linear variable. Finally, although a large cohort of HDV patients was studied, the absolute number of IgM negative patients was still limited. On the other hand, particular strengths of the study are the large sample size, the very well defined cohort of patients recruited in an international multicenter trial, and validation of key findings in an independent cohort.

In summary, our date show that anti-HDV IgM testing is a relatively easy and robust marker which can provide important clinical information as IgM values were associated with disease activity and clinical long-term outcome. We suggest that IgM testing should be introduced in the routine clinical workup of hepatitis delta patients.

## Supporting Information

Figure S1 **Evaluation of the anti-HDV IgM assay indicated deviations of the optical density values based on variation in time.**
(TIF)

Table S1 **All measured cytokines, chemokines and angiogenic factors associated with the groups of anti-HDV IgM based on ANOVA analysis.**
(DOCX)
