# Supplementary figures and images for: Anti-HDV IgM as a Marker of Disease Activity in Hepatitis Delta
Source: PLoS One. 2014 Jul 29;9(7):e101002. doi: 10.1371/journal.pone.0101002 (PMC4114528; doi:10.1371/journal.pone.0101002)

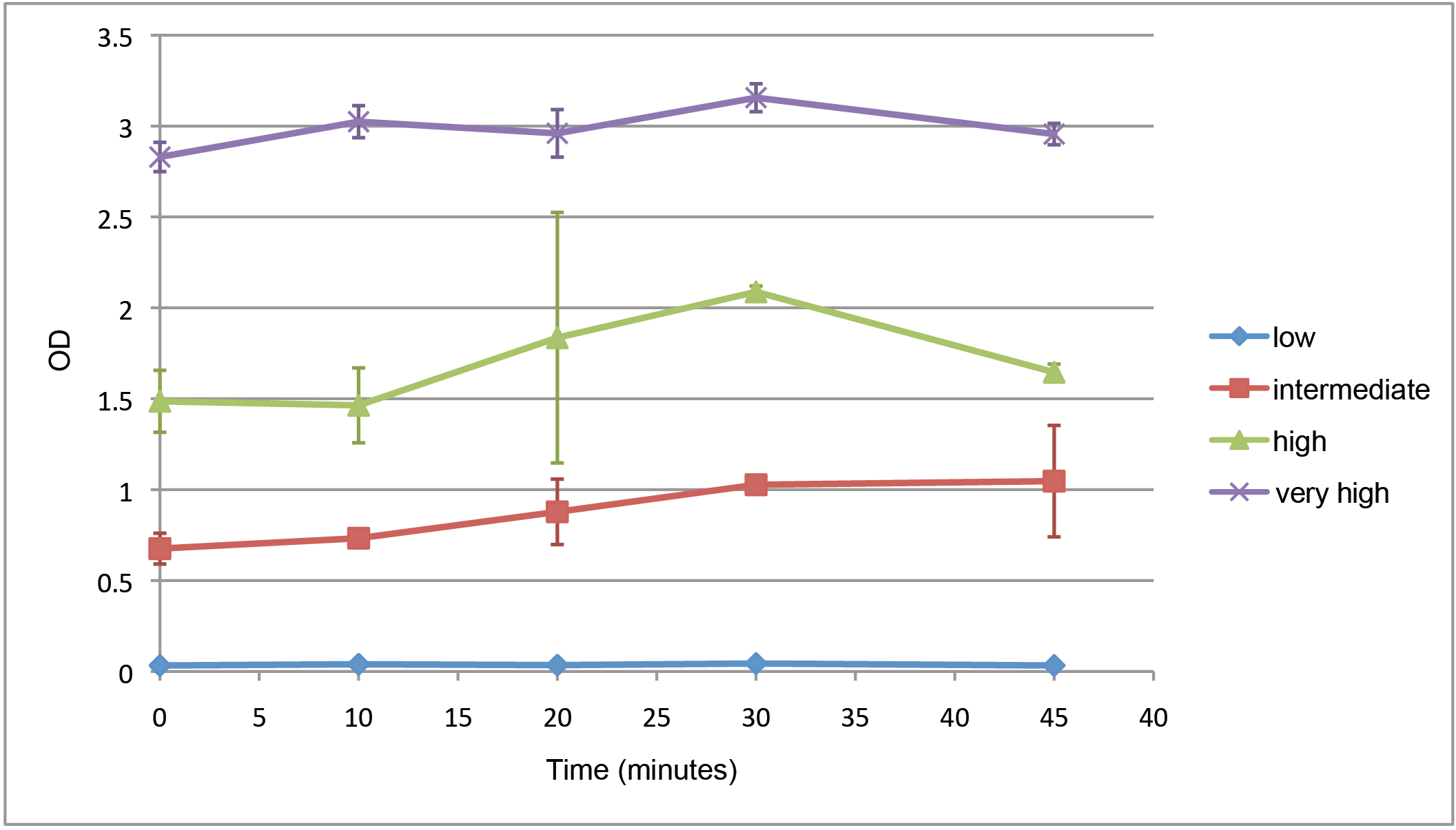

Supplement: Figure S1 — Evaluation of the anti-HDV IgM assay indicated deviations of the optical density values based on variation in time. (TIF) [file pone.0101002.s001.tif]
