# Supplementary material for: Anti-HDV IgM as a Marker of Disease Activity in Hepatitis Delta
Source: PLoS One. 2014 Jul 29;9(7):e101002. doi: 10.1371/journal.pone.0101002 (PMC4114528; doi:10.1371/journal.pone.0101002)
Supplement: Table S1 — All measured cytokines, chemokines and angiogenic factors associated with the groups of anti-HDV IgM based on ANOVA analysis. (DOCX) [file pone.0101002.s002.docx]

Table S1.

*All measured cytokines, chemokines and angiogenic factors associated with the groups of anti-HDV IgM based on ANOVA analysis.*

|  | ***p-value*** |
| --- | --- |
| **IL-1β** | **0.05** |
| IL-1Rα | 0.08 |
| IL-2 | 0.12 |
| IL-4 | 0.08 |
| IL-5 | 0.30 |
| IL-6 | 0.12 |
| IL-7 | 0.33 |
| **IL-8** | **0.04** |
| IL-9 | 0.55 |
| IL-10 | 0.38 |
| IL-13 | 0.25 |
| IL-12 (p70) | 0.13 |
| IL-15 | 0.15 |
| **IL-17** | **0.04** |
| IL-18 | 0.17 |
| Eotaxin | 0.16 |
| FGF-β | 0.06 |
| G-CSF | 0.48 |
| GM-CSF | 0.19 |
| IFN-γ | 0.09 |
| **IP-10** | **0.02** |
| **MCP-1** | **0.04** |
| MIP-1α | 0.07 |
| PDGF-ββ | 0.16 |
| MIP-1β | 0.79 |
| RANTES | 0.64 |
| TNF-α | 0.06 |
| VEGF | 0.20 |
| **IL-1α** | **0.05** |
| **IL-2Rα** | **<0.01** |
| IL-3 | 0.06 |
| IL-12 (p40) | 0.07 |
| **IL-16** | **0.03** |
| CTACK | 0.27 |
| GRO-α | 0.27 |
| HGF | 0.14 |
| ICAM-1 | 0.83 |
| IFN-α2 | 0.25 |
| **LIF** | **0.04** |
| MCP-3 | 0.09 |
| **M-CSF** | **0.05** |
| MIF | 0.20 |
| MIG | 0.25 |
| **β-NGF** | **0.05** |
| **SCF** | **0.03** |
| SCGF-β | 0.11 |
| **SDF-1α** | **0.03** |
| **TNF-β** | **0.05** |
| TRAIL | 0.06 |
| VCAM-1 | 0.18 |

* all significant parameters are listed in bold
